# Orthostatic hypotension in Parkinson’s disease: effects on clinical features and disease severity-a systematic review and meta-analysis

**DOI:** 10.3389/fnagi.2025.1612960

**Published:** 2025-07-17

**Authors:** Hui Wang, Chi Zhang, Dongxun Xu

**Affiliations:** ^1^Department of Neurology, Sichuan Taikang Hospital, Chengdu, Sichuan, China; ^2^Department of Medical Psychology, Daping Hospital, Army Medical University, Chongqing, China

**Keywords:** Parkinson’s disease, depression, motor symptoms, non-motor symptoms, disease progress

## Abstract

**Background:**

Parkinson’s disease (PD) is often associated with orthostatic hypotension (OH). However, research examining the relationship between OH and PD has yielded inconsistent results. This study conducts a meta-analysis to determine the associations between OH and clinical characteristics in individuals with PD.

**Methods:**

A systematic review and meta-analysis were performed by searching for studies related to PD and OH in the PubMed, Web of Science, Embase, and Cochrane databases. Data were pooled as necessary to calculate the odds ratios (OR) and weighted mean differences (WMD) with 95% confidence intervals (CI) for OH in PD patients. Heterogeneity was assessed using the I^2^ statistic. Meta-regression was conducted to detect the potential influences of disease duration on the differences in clinical features between PD patients with and without OH.

**Results:**

A total of 26 articles involving 3,992 patients with Parkinson’s disease were included in our study. Patients with PD and OH were significantly older at the time of examination compared to those without OH (WMD 2.94 years, 95% CI 1.91–3.97 years; I^2^ = 64.1%). PD patients with OH had a significantly longer disease duration than those without OH (WMD 0.73, 95% CI 0.32–1.14). Furthermore, PD patients with OH exhibited significantly lower Mini-Mental State Examination (MMSE) and Montreal Cognitive Assessment (MoCA) scores than those without OH (WMD of − 0.99, 95% CI of −1.91 to −0.07; WMD of −1.86, 95% CI of −2.67 to −1.04). There were no significant differences in gender distribution, Hamilton Anxiety Rating Scale (HAMA) or Hamilton Depression Rating Scale (HAMD) scores among Parkinson’s disease patients with or without OH.

**Conclusion:**

Patients with PD and OH tend to be older at the time of examination, exhibit a longer disease duration, and demonstrate more severe disease manifestations along with greater cognitive impairment compared to PD patients without OH.

**Systematic review registration:**

https://www.crd.york.ac.uk/prospero/, identifier CRD420251025263.

## Introduction

Parkinson’s disease (PD) represents a progressive neurodegenerative disorder that is characterized by the pathology of Lewy bodies in both the central and peripheral nervous systems (Boeve, 2013). This disorder manifests primarily as a movement impairment, featuring symptoms such as tremors, rigidity, slowed movements (bradykinesia), and difficulties with posture stability. Moreover, patients with PD often experience various non-motor symptoms, including issues related to mood, sleep, cognition, autonomic dysfunction and olfactory senses (Morley and Hurtig, 2010). These non-motor symptoms can significantly impact the quality of life for those affected, leading to complications like constipation, irregular urination, sexual dysfunction, excessive perspiration, and orthostatic hypotension (OH) (Chaudhuri et al., 2006).

Orthostatic hypotension is frequently observed among individuals with PD, presenting in about 27.7% of PD patients (Mu et al., 2020). It is defined by a decrease of at least 20 mmHg in systolic blood pressure or a reduction of at least 10 mmHg in diastolic blood pressure within a 3 min interval after standing up or during a head-up tilt (Freeman et al., 2011). Recent research has shed light on various factors, such as age, disease severity, medication use, and hypertension, which may increase the likelihood of experiencing OH (Klanbut et al., 2018). Prior investigations suggest that OH could act as an early clinical indicator of Parkinson’s disease, characterized by initial autonomic dysfunction, where peripheral nerves and the heart are impacted before any central nervous system involvement occurs (Mei et al., 2024). Patients with PD exhibiting OH may experience a quicker onset of multisystem complications (Mei et al., 2024). Additionally, the presence of OH in PD is often associated with exacerbated non-motor symptoms, including rapid eye movement sleep behavior disorder (RBD) and cognitive decline (Katsi et al., 2021; Schapira et al., 2017). Early detection of OH is crucial, as its emergence could indicate preliminary signs of broader nervous system dysfunction.

In recent years, investigations into the impact of OH on the severity of disease in PD patients have seen a significant rise compared to prior research; however, the outcomes of these studies have been inconsistent. Certain investigations reveal that PD patients experiencing OH are generally older, have a prolonged disease duration, and demonstrate more pronounced motor and cognitive symptoms than those without OH (Mei et al., 2024; Yin et al., 2022; Yoo et al., 2021b). In contrast, other research has found no noteworthy differences in age, duration of the disease, or the severity of motor symptoms, cognition, and mood between Parkinson’s disease patients who do and do not have OH (Nakamura et al., 2020; Umehara et al., 2021; Wu et al., 2021). This highlights the importance of integrating earlier studies for the purpose of meta-analysis.

This topic is significant because OH is a treatable condition that may represent a modifiable risk factor for the acceleration of PD symptoms. This paper presents a comprehensive systematic review and meta-analysis of research conducted over the past 25 years on the effects of OH on PD.

## Methods

### Searching strategy

This meta-analysis was registered with the International Prospective Register of Systematic Reviews (PROSPERO, No. CRD420251025263) and was conducted in accordance with the guidelines established by the Preferred Reporting Items for Systematic Reviews and Meta-Analyses (PRISMA) (Moher et al., 2009). The Cochrane Collaboration definition for systematic review and meta-analysis was strictly followed. Two authors (H. Wang and C. Zhang) independently searched Medline via PubMed, Web of Science, Embase via embase.com, and Cochrane databases for original published studies on the clinical manifestations of PD patients with or without OH. Inclusion criteria were studies published in English between January 2000 and March 2025. The search string was as follows: (“Hypotension, Orthostatic” OR “Hypotension, Postural” OR “Postural Hypotension” OR “Orthostatic Hypotension”) AND (“Idiopathic Parkinson’s Disease” OR “Lewy Body Parkinson’s Disease” OR “Parkinson Disease” OR “Parkinson’s Disease, Idiopathic” OR “Parkinson’s Disease, Lewy Body” OR “Paralysis Agitans” OR “Parkinson’s Disease” OR “Idiopathic Parkinson Disease” OR “Lewy Body Parkinson Disease” OR “Primary Parkinsonism” OR “Parkinsonism, Primary” OR “Parkinson Disease, Idiopathic” OR “Parkinson Disease”) (Supplementary Table 1).

### Study selection criteria

Articles were initially screened based on their titles and abstracts, with full text consulted when necessary. Patients were diagnosed with OH according to a fall of ≥ 20 mmHg systolic or ≥ 10 mmHg diastolic blood pressure upon 1–3 min of active standing or head up tilt (Freeman et al., 2011).

The criteria for inclusion were defined as follows: (1) studies had to be original research with designs such as cross-sectional, case-control, or cohort; (2) the main outcome of focus was OH; (3) sufficient data needed to be present to evaluate variations in the severity of non-motor symptoms linked to PD.

The criteria for exclusion included: (1) reviews, editorials, letters, conference abstracts, or case reports; (2) studies that were solely centered on characteristics of OH, pathogenic mechanisms, or management strategies for PD related to OH; (3) comparisons made between PD and other synucleinopathies; (4) studies lacking adequate data for a meta-analysis; (5) articles written in languages other than English; and (6) research involving animal subjects.

Discrepancies regarding article inclusion were resolved by a third author, D. Xu.

### Data extraction and study quality assessment

The data extracted from the original articles included the surname of the first author, the publication year, the country, the sample size, the diagnostic criteria for PD, the method of assessing OH, the mean age of patients, their sex, and the duration of the disease. For longitudinal studies, only baseline data were extracted.

The quality of the included studies was assessed using the Newcastle-Ottawa Scale (NOS) for case-control and cohort studies, along with the Agency for Healthcare Research and Quality (AHRQ) guidelines for cross-sectional studies (Rostom et al., 2004; Wells et al., 2012). Any discrepancies were resolved through consensus among all authors.

### Statistical analysis

The STATA software version 16.0 was used for statistical analysis. Odds ratio (OR), weighted mean difference (WMD), or standardized mean difference (SMD) with 95% confidence intervals (95% CI) were used to report pooled results on dichotomous and continuous variables. A *p*-value equal to or less than 0.05 was considered statistically significant. The potential influences of disease duration on the differences of clinical features between PD patients with and without OH were detected by univariate meta-regression analyses. The heterogeneity across studies was evaluated using Cochrane’s I^2^ values. I^2^ > 75% was defined as high heterogeneity, 50% < I^2^ < 75% as moderate heterogeneity, 25% < I^2^ < 50% as low heterogeneity, and I^2^ < 25% as homogeneity. We used a fixed-effects model to meta-analyze data showing homogeneity and low heterogeneity, and a random-effects model to analyze data classified as moderate or high heterogeneity. A sensitivity analysis was performed to detect potential sources of heterogeneity. We used the one by one elimination method on STATA to perform sensitivity analyses to detect potential sources of heterogeneity, and when the number of studies was ≥ 10 and < 20, we used the Egger’s test to detect publication bias, whereas when the number of studies was < 10 or ≥ 20, we used the Begg’s or Egger’s test to detect publication bias.

## Results

**FIGURE 1:**
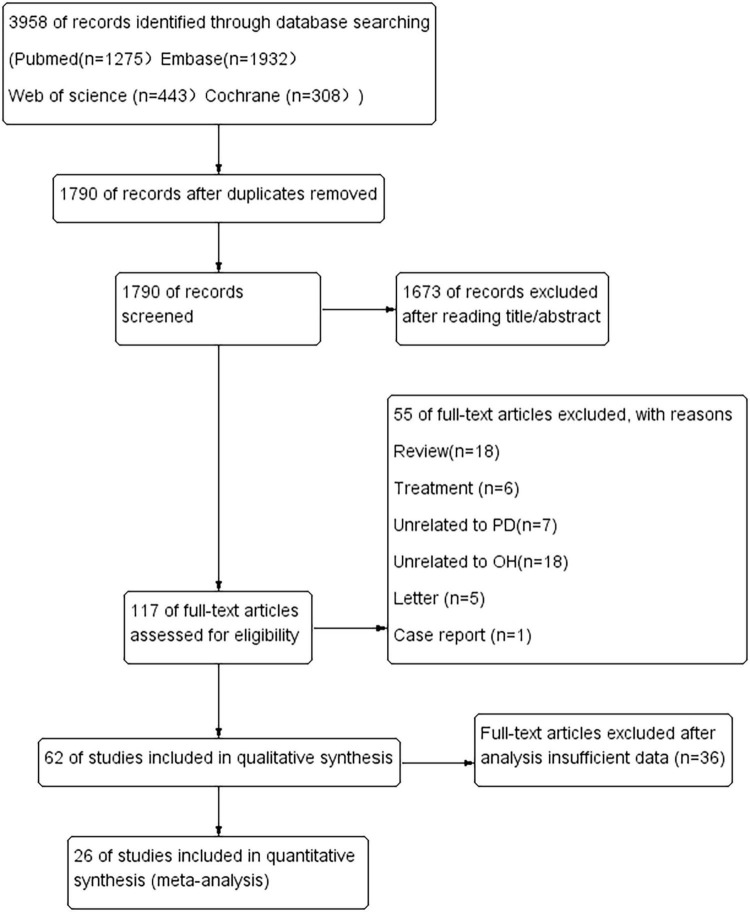
Flow diagram of systematic literature searching.

The literature search identified 3,958 potentially relevant articles (Figure 1). After the removal of duplicates, 1,790 records were reviewed, resulting in the exclusion of 2,168 during the title and abstract screening phase. Subsequently, 117 full-text articles were assessed for eligibility, of which 55 were excluded for various reasons: 18 were reviews, six were studies focused on treatment, seven were unrelated to PD, 18 were unrelated to OH, five were letters, one was a case report, and 36 had insufficient data.

Ultimately, our review included 26 articles, comprising a total of 3,992 patients with PD. Among these studies, 16 were conducted in Asia, four in America, and six in Europe. A meta-analysis was performed on all PD patients, taking into account factors such as age, sex, disease duration, non-motor symptoms (including cognitive and mood-related aspects), and the Unified Parkinson’s Disease Rating Scale (UPDRS-III).

### Differences in age, gender, and disease duration between PD patients with or without OH

**FIGURE 2:**
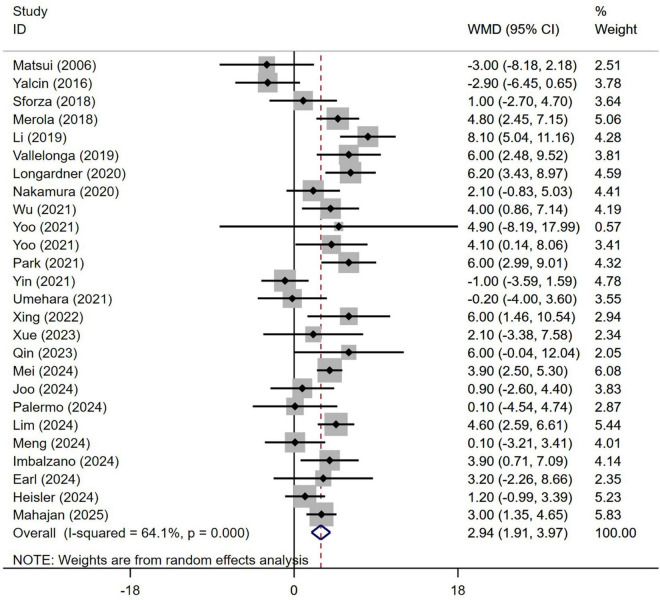
Forest plot showing weighted mean difference (WMD) in age between Parkinson’s disease patients with or without orthostatic hypotension.

Subgroup analysis based on age included 3,992 patients (Table 1). PD patients with OH were significantly older at the time of examination compared to those without OH (WMD 2.94 years, 95% CI 1.91–3.97; I^2^ = 64.1%; Figure 2). There was no evidence of publication bias, as Begg’s test yielded a non-significant result (Supplementary Figure 1A). The heterogeneity of this study was moderate (I^2^ = 65.6%). Sensitivity analysis indicated that the results remained unchanged (Supplementary Figure 1B).

**TABLE 1:** Characteristics of studies included in the meta-analyses.

References	Country	Diagnosis criteria for PD	Diagnostic OH method	Sample size	Mean age of patients with OH/without OH	AHRQ/NOS score
Matsui et al., 2006	Japan	PDSBB	DC	40	70.0 ± 8.7/73.0 ± 7.7	6
Yalcin et al., 2016	Turkey	PDSBB	DC	84	71.3 ± 8.8/74.2 ± 7.1	6
Sforza et al., 2018	Italy	PDSBB	DC	28	73 ± 5/72 ± 5	6
Merola et al., 2018	United States	PDSBB	DC	319	74.4 ± 9.5/69.6 ± 10.2	6*
Li et al., 2019	China	PDSBB	DC	150	70.2 ± 8.1/62.1 ± 10.5	7
Vallelonga et al., 2019	Italy	PDSBB	DC	113	68 ± 8/62 ± 11	7
Longardner et al., 2020	United Kingdom	PDSBB	DC	226	71.0 ± 9.3/64.8 ± 10.8	6
Nakamura et al., 2020	Japan	PDSBB	DC	128	68.6 ± 7.7/66.5 ± 6.5	6
Wu et al., 2021	China	PDSBB	DC	158	67.2 ± 8.0/63.2 ± 12.1	7
Yoo et al., 2021b	Korea	PDSBB	DC	221	72.6 ± 7.7/67.7 ± 10.7	6*
Yoo et al., 2021a	Korea	PDSBB	DC	73	73.8 ± 7.1/69.7 ± 9.1	6
Park et al., 2021	Korea	PDSBB	DC	70	65 ± 5/59 ± 5	6*
Yin et al., 2022	China	PDSBB	DC	116	66 ± 5.5/67 ± 7.5	6
Umehara et al., 2021	Japan	PDSBB	DC	81	70.5 ± 7.6/70.7 ± 9.8	6
Xing et al., 2022	China	PDSBB	DC	90	63.7 ± 7.7/57.7 ± 11.1	7
Xue et al., 2023	China	PDSBB	DC	31	60.7 ± 8.2/58.6 ± 7.0	6
Qin et al., 2023	China	PDSBB	DC	80	66 ± 12/60 ± 8	6*
Mei et al., 2024	China	PDSBB	DC	607	64.1 ± 7.7/60.2 ± 9.7	7
Joo et al., 2024	Korea	PDSBB	DC	31	69.6 ± 3.6/68.7 ± 6.3	6
Palermo et al., 2024	Italy	PDSBB	DC	22	71.2 ± 5.7/71.1 ± 5.4	6
Lim et al., 2024	Malaysia	PDSBB	DC	318	69.1 ± 8.2/64.5 ± 9.7	6
Meng et al., 2024	China	PDSBB	DC	171	65.3 ± 11.4/65.2 ± 10.6	7
Imbalzano et al., 2024	Italy	PDSBB	DC	82	63.5 ± 6.8/59.6 ± 7.0	7
Earl et al., 2024	United States	PDSBB	DC	40	75.0 ± 4.0/71.8 ± 10.9	6*
Heisler et al., 2024	United States	PDSBB	DC	250	69.5 ± 7.7/68.3 ± 9.0	7
Mahajan et al., 2025	United States	PDSBB	DC	463	69.2 ± 7.6/66.2 ± 10.2	7
Total				3,992		

*Evaluating cohort or case-control studies quality using NOS. AHRQ, the Agency for Healthcare Research and Quality guideline; DC, diagnostic criteria; NOS, Newcastle-Ottawa Scale; OH, orthostatic hypotension; PDSBB, UK Parkinson’s Disease Society Brain Bank clinical diagnosis criteria. DC is defined as a fall of ≥ 20 mmHg systolic or ≥ 10 mmHg diastolic blood pressure upon 1–3 min of active standing or head up tilt according to reference 1.

**FIGURE 3:**
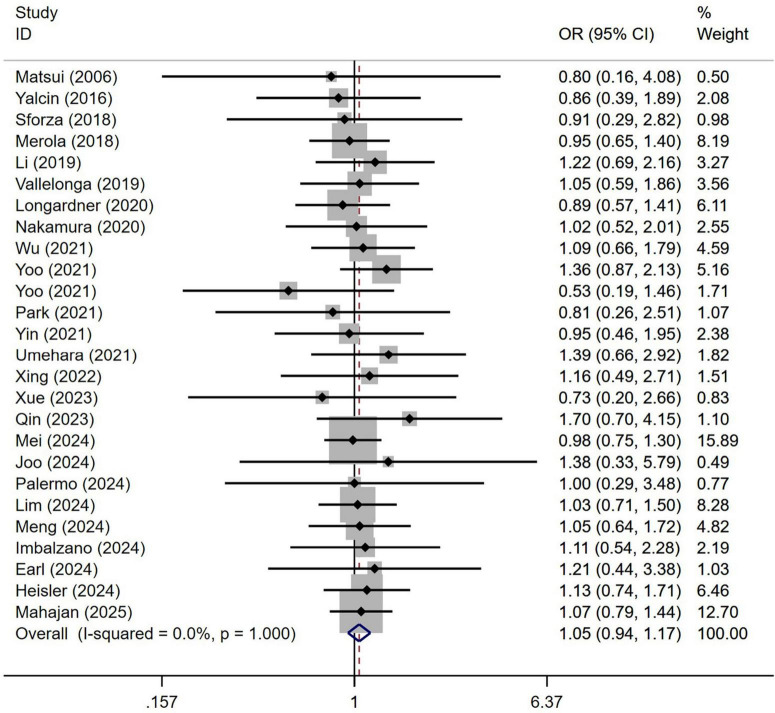
Forest plot showing odds ratio (OR) in sex between Parkinson’s disease patients with or without orthostatic hypotension.

A total of 3,992 patients with PD from 26 studies were included in the analysis by sex. No significant difference in sex distribution was observed between PD patients with or without OH (Figure 3). Our study demonstrated homogeneity (I^2^ = 0%). Begg’s test did not reveal significant publication bias (Supplementary Figure 2).

**FIGURE 4:**
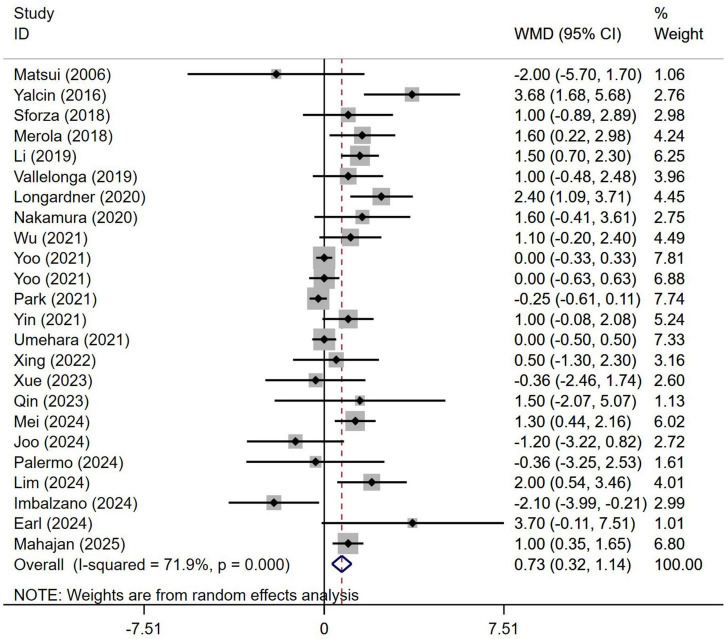
Forest plot showing weighted mean difference (WMD) in disease duration between Parkinson’s disease patients with or without orthostatic hypotension.

A meta-analysis of the disease duration in 3,557 patients with PD from 24 studies indicated that the disease duration in PD patients with OH was significantly longer than in those without OH (WMD 0.73, 95% CI 0.32–1.14; Figure 4). Begg’s test did not show significant publication bias (Supplementary Figure 3A). The heterogeneity of this study was moderate (I^2^ = 71.9%). Sensitivity analysis confirmed that the results remained unchanged (Supplementary Figure 3B).

### Differences in non-motor symptoms between PD patients with or without OH

**FIGURE 5:**
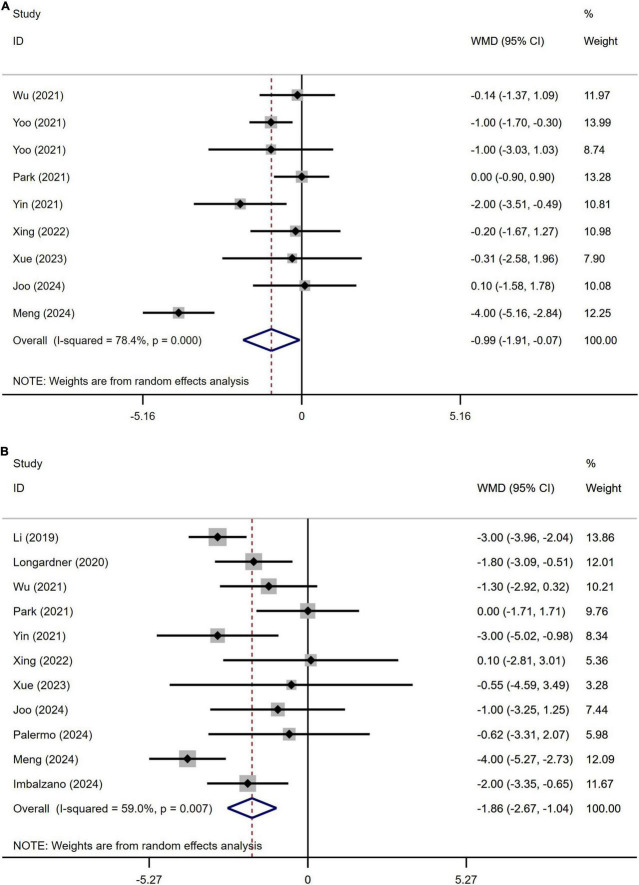
Forest plot showing weighted mean difference (WMD) in the Mini-Mental State Examination (MMSE) **(A)** and the Montreal Cognitive Assessment (MoCA) **(B)** scores between Parkinson’s disease patients patients with or without orthostatic hypotension.

A meta-analysis of the cognitive abilities assessed using the Mini-Mental State Examination (MMSE) score in 961 patients with PD from nine studies indicated that the MMSE scores in PD patients with OH was significantly lower than in those without OH (WMD −0.99, 95% CI −1.91 to −0.07; Figure 5A). Begg’s test did not show significant publication bias (Supplementary Figure 4A). The heterogeneity of this study was high (I^2^ = 78.4%). Sensitivity analysis confirmed that the results remained unchanged (Supplementary Figure 4B).

A meta-analysis of the cognitive abilities assessed using the Montreal Cognitive Assessment (MoCA) score in 1147 patients with PD from 11 studies indicated that MoCA score in PD patients with OH was significantly lower than in those without OH (WMD −1.86, 95% CI −2.67 to −1.04; Figure 5B). Egger’s test did not show significant publication bias (Supplementary Figure 5A). The heterogeneity of this study was moderate (I^2^ = 59.0%). Sensitivity analysis confirmed that the results remained unchanged (Supplementary Figure 5B).

**FIGURE 6:**
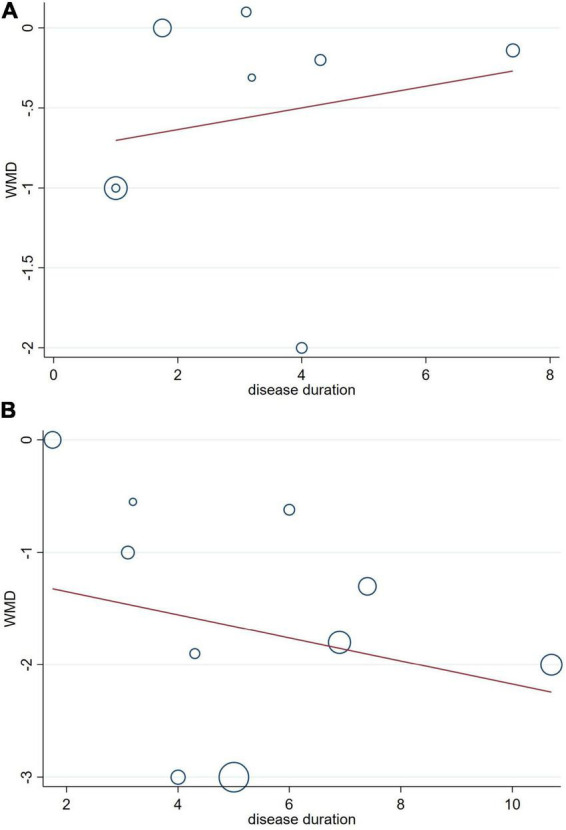
Forest plot showing meta-regression modeling of the ini-Mental State Examination (MMSE) **(A)** and the Montreal Cognitive Assessment (MoCA) **(B)** scores in Parkinson’s disease with orthostatic hypotension patients adjusted for disease duration.

The potential influences of disease duration on the differences of cognition between PD patients with and without OH were detected by univariate meta-regression analyses. Disease duration was not significantly related to the MMSE and MoCA scores (Figures 6A, B).

**FIGURE 7:**
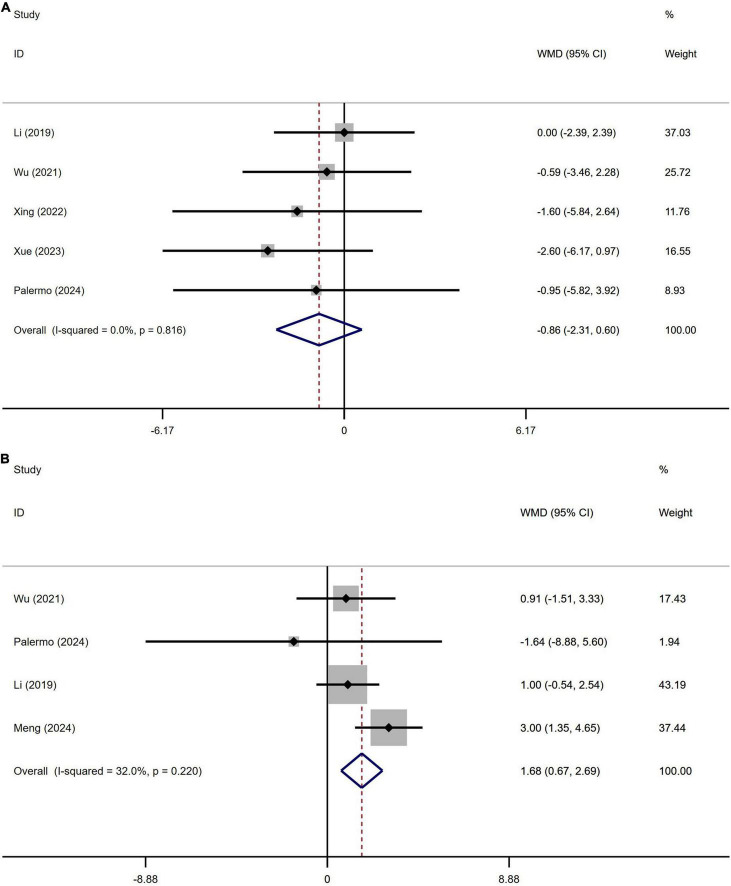
Forest plot showing weighted mean difference (WMD) in the Hamilton Depression Scale (HAMD) **(A)** and Hamilton Anxiety Rating Scale (HAMA) **(B)** between Parkinson’s disease patients with or without orthostatic hypotension.

A total of 622 patients with PD from five studies were included in the analysis of Hamilton Depression Scale (HAMD) scores. No significant difference in HAMD scores was observed between PD patients with or without OH (Figure 7A). Begg’s test did not reveal significant publication bias (Supplementary Figure 6A). This study demonstrated homogeneity (I^2^ = 0%).

A total of 501 patients with PD from four studies were included in the analysis of Hamilton Depression Scale (HAMD) scores. No significant difference in HAMD scores was observed between PD patients with or without OH (Figure 7B). Begg’s test did not reveal significant publication bias (Supplementary Figure 6B). The heterogeneity of this study was low (I^2^ = 32%).

### Differences in the UPDRS-III between PD patients with or without OH

**FIGURE 8:**
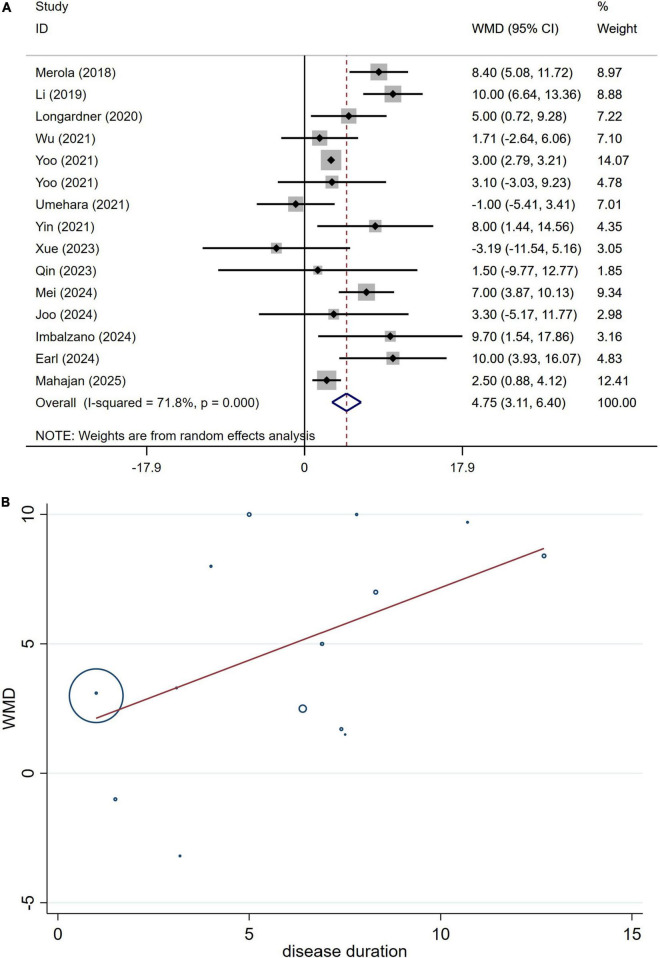
Forest plot showing weighted mean difference (WMD) **(A)** and meta-regression modeling **(B)** of UPDRS-III scores between Parkinson’s disease patients with or without orthostatic hypotension.

A total of 2,664 patients with PD from 15 studies were included in the analysis of UPDRS Part III section scores. The results indicated that the UPDRS Part III section scores in PD patients with OH were significantly higher than those in PD patients without OH (WMD 4.75, 95% CI 3.31–6.40; Figure 8A). Begg’s test did not reveal significant publication bias (Supplementary Figure 7A). The heterogeneity of this study was moderate (I^2^ = 71.8%). Sensitivity analysis yielded consistent results (Supplementary Figure 7B).

The potential influences of disease duration on the differences of the UPDRS-III section scores between PD patients with and without OH were detected by univariate meta-regression analyses. Disease duration was not significantly related to the UPDRS-III section scores (Figure 8B).

## Discussion

This meta-analysis examined motor and non-motor symptoms in individuals with PD, comparing groups with and without OH.

Prior research has produced inconsistent results concerning the variability associated with factors such as age, disease duration, and cognitive assessments like UPDRS-III and MMSE or MoCA scores among individuals living with PD, in both those with and without OH. Certain studies suggest that PD patients experiencing OH are generally older, have an extended disease duration, and display more pronounced motor and cognitive symptoms compared to those without OH (Mei et al., 2024; Yin et al., 2022; Yoo et al., 2021b). In contrast, alternative research has found no meaningful distinctions in age, duration of the disease, or severity of motor and cognitive symptoms between PD patients with OH and those without (Nakamura et al., 2020; Umehara et al., 2021; Wu et al., 2021). The possible reasons for this phenomenon are as follows: First, variations in ethnicity may influence the results, necessitating multi-center studies encompassing diverse ethnic groups and regions to validate our hypothesis. Second, the studies in question feature differing sample sizes; for instance, some studies have only 22 participants (Palermo et al., 2024), while others include 463 participants (Mahajan et al., 2025). The findings from studies with smaller sample sizes may be unreliable due to insufficient data. To resolve this contradiction, we undertook a meta-analysis that included all pertinent studies on the effects of OH on PD, thereby enhancing the overall sample size for more reliable conclusions. Our findings indicated that at the point of assessment, PD patients suffering from OH were considerably older than their peers without OH (Figure 2). Furthermore, those diagnosed with PD and OH had a significantly longer disease duration compared to individuals without OH (Figure 4). Additionally, PD patients with OH demonstrated markedly elevated UPDRS-III scores, which signify more severe motor symptoms (Figure 8). Moreover, given that disease duration is a critical factor influencing the severity of motor symptoms in PD patients, we performed univariate meta-regression analyses to ascertain the potential impacts of disease duration on the differences in UPDRS-III scores between patients with and without OH. The findings indicated that disease duration did not have a significant influence on the statistical results related to the differences in UPDRS-III scores (Figure 8). This suggests that as the disease advances, motor symptoms become more pronounced in PD patients with OH compared to those without it.

The fundamental pathological mechanism underlying PD primarily consists of the degeneration and necrosis of dopamine neurons located in the nigrostriatal pathway, this neurodegeneration is significantly connected to the formation and aggregation of Lewy bodies, which are abnormal protein accumulations found in the brains of PD patients (Jiang et al., 2017; Sulzer and Edwards, 2019). Additionally, research indicates that similar pathological alterations are not restricted to the substantia nigra alone; they are also present in various other regions of the central nervous system (CNS). These areas include the dorsal nucleus of the thalamus, insular cortex, dorsal nucleus of the vagus nerve, sympathetic ganglia, and the enteric nervous system, the involvement of these regions is particularly relevant because they are linked to autonomic dysfunction, which can lead to a range of symptoms experienced by individuals with PD (Wang et al., 2019; Zhang et al., 2017). Dysautonomia in Parkinson’s disease results from the degeneration of both central and peripheral sympathetic pathways, impaired baroreflexes, and reduced norepinephrine-mediated vasoconstriction. Peripheral autonomic failure may play a more significant role in patients with Parkinson’s disease who are experiencing OH (Coon et al., 2018; Cuenca-Bermejo et al., 2021; Mei et al., 2024). Furthermore, it is important to note that medications and cardiac denervation can further exacerbate this condition (Rivasi et al., 2020). Building on the findings of our meta-analysis and the established pathophysiological framework outlined above, we put forward the hypothesis that an extended duration of Parkinson’s disease is associated with a higher probability of developing OH. Moreover, the occurrence of OH among PD patients may serve as an indicator of extensive central and peripheral nervous system damage, suggesting a more accelerated rate of neurodegenerative processes. The results of previous longitudinal trial indicates that OH may be associated with accelerated disease progression in patients with Parkinson’s disease, thereby supporting our hypothesis (Yoo et al., 2021b).

The results of this meta-analysis indicate that there is no significant difference in the HAMA and HAMD scores between PD patients with OH and those without. These findings align with previous studies (Li et al., 2019; Meng et al., 2024; Palermo et al., 2024; Wu et al., 2021; Xing et al., 2022; Xue et al., 2023). However, patients with PD who experience OH exhibit lower scores on the MMSE and the MoCA compared to those without OH (Figure 5). This suggests that PD patients with OH may experience more severe cognitive dysfunction. Additionally, we conducted a meta-regression analysis to control for the influence of disease duration on these outcomes. Prior research has demonstrated that OH is linked to overall cognitive decline, memory impairment, and executive dysfunction, particularly in the elderly population (Duval et al., 2023). This association may be attributed to the transient reduction in cerebral blood flow caused by OH, especially in older individuals with compromised cerebral autoregulation, which could precipitate white matter hyper-signaling or cortical atrophy (Starmans et al., 2024). Based on the results of our meta-analysis, it appears that while OH impacts cognitive function in patients with Parkinson’s disease, it may not influence the rate of cognitive decline. However, it is important to note that our study primarily consists of cross-sectional studies, and for longitudinal studies, only baseline data were extracted. Consequently, it remains uncertain whether cognitive decline is a direct consequence of OH or if OH exacerbates the progression of Parkinson’s disease, thereby accelerating the onset of cognitive dysfunction. However, it is worth noting that previous longitudinal studies have indicated that OH may be associated with the accelerated disease progression of cognitive dysfunction in patients with Parkinson’s disease (Yoo et al., 2021b). Therefore, further multicenter longitudinal studies are necessary to validate this conclusion.

This study acknowledges several limitations. Firstly, the diagnosis of neurodegenerative diseases was primarily based on clinical assessments. While these assessments are essential for identifying such conditions, they may not always provide the highest level of diagnostic accuracy. The reliance on clinical judgment might lead to variations in diagnosis, which could affect the study’s overall conclusions regarding the prevalence and characteristics of these diseases. Secondly, levodopa equivalent daily dose with OH is missing, since patients with longer disease duration are likely taking higher doses of levodopa, which could lower the pressure. Thirdly, it is essential to recognize that OH can present in two distinct forms: Classical OH and delayed OH. Classical OH is traditionally defined as a sustained decrease in systolic blood pressure of ≥ 20 mmHg and/or diastolic blood pressure of ≥ 10 mmHg within 3 min of standing. In contrast, delayed OH is characterized by a progressive drop in blood pressure that exceeds the initial change after 3 min (Yoo et al., 2021b). Previous studies have predominantly focused on Classical OH patients, potentially introducing a bias into the findings. Finally, the scope of our meta-analysis was limited to studies published in English. This restriction may have inadvertently introduced a language bias, as valuable research published in other languages was excluded from consideration. Such a limitation could affect the comprehensiveness of the findings and potentially skew the interpretation of the results.

## Conclusion

Patients with PD who experience OH are typically older at the time of examination, have a longer disease duration, and display more severe manifestations of the disease, along with greater cognitive impairment compared to those without OH. The presence of OH in PD patients may indicate extensive damage to both the central and peripheral nervous systems, suggesting a potentially accelerated neurodegenerative process.

## Data Availability

The original contributions presented in this study are included in this article/Supplementary material, further inquiries can be directed to the corresponding author.
