# Supplementary figures and images for: Orthostatic hypotension in Parkinson’s disease: effects on clinical features and disease severity-a systematic review and meta-analysis
Source: Front Aging Neurosci. 2025 Jul 17;17:1612960. doi: 10.3389/fnagi.2025.1612960 (PMC12310585; doi:10.3389/fnagi.2025.1612960)

Fig.1.A


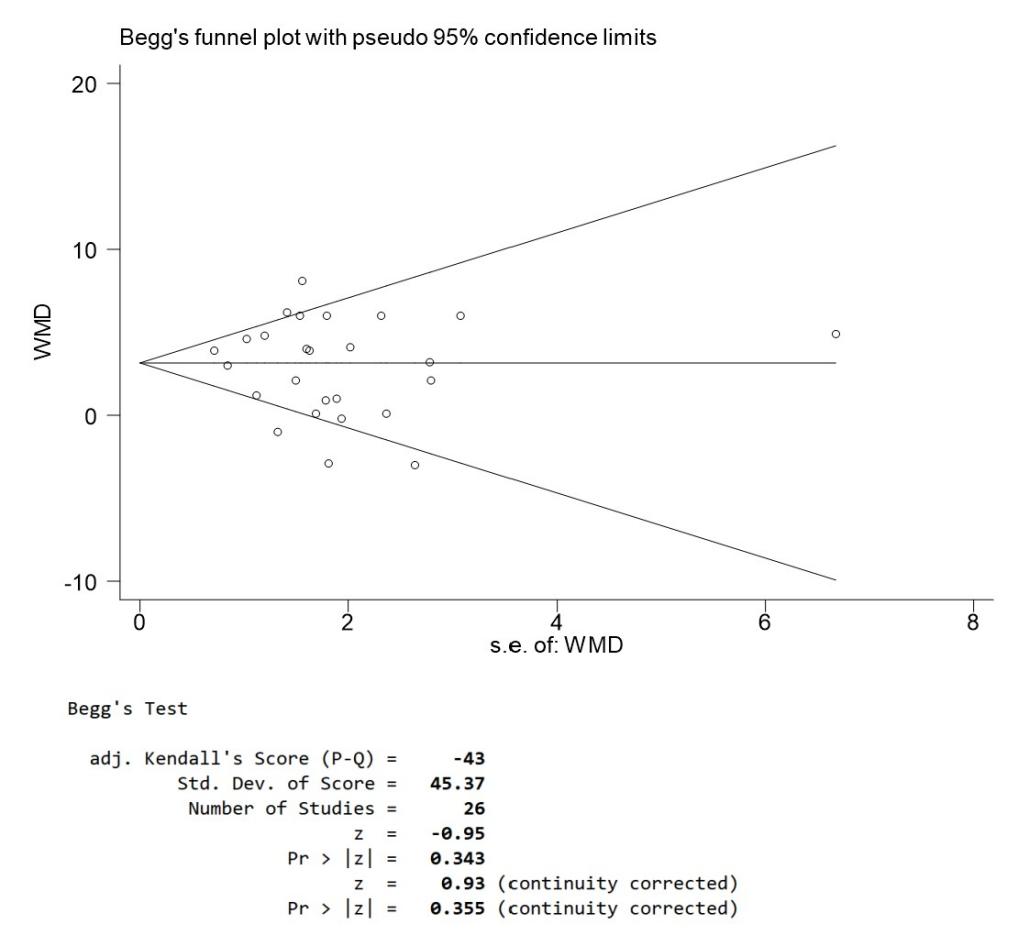


Fig.1.B


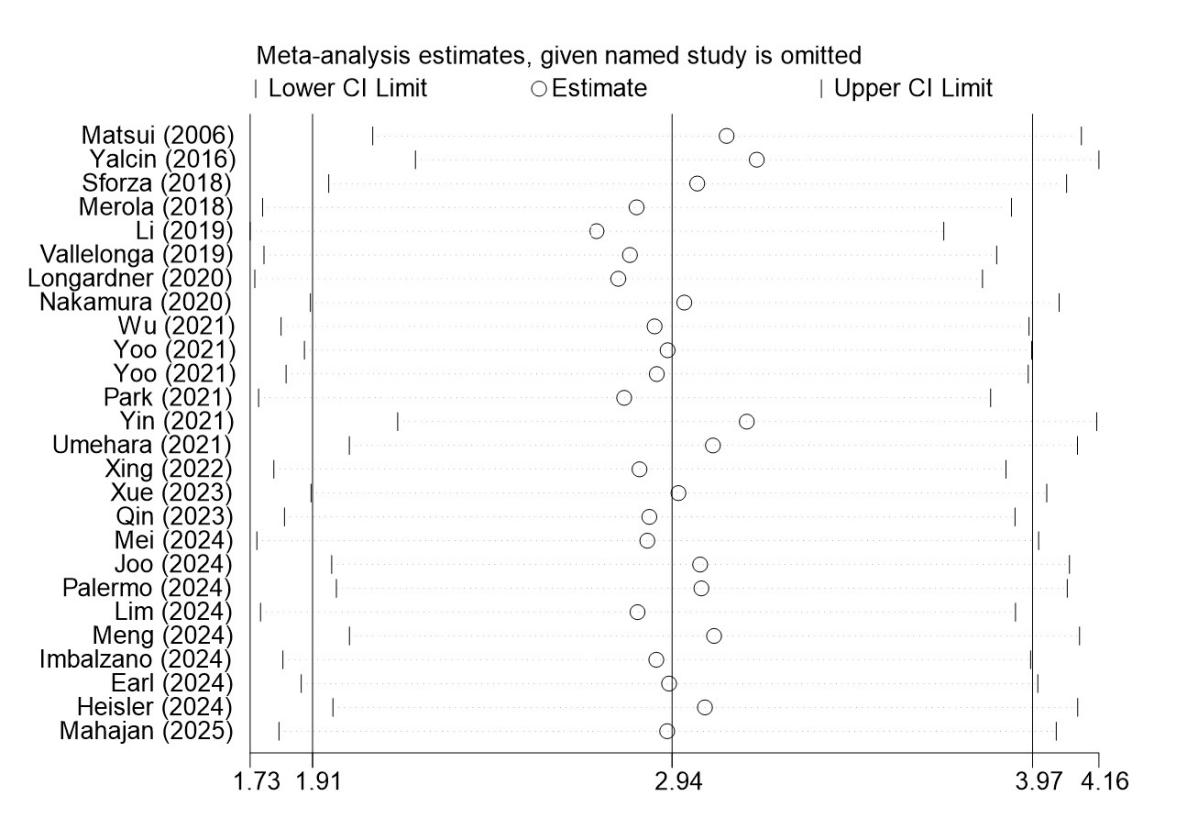


Fig.2


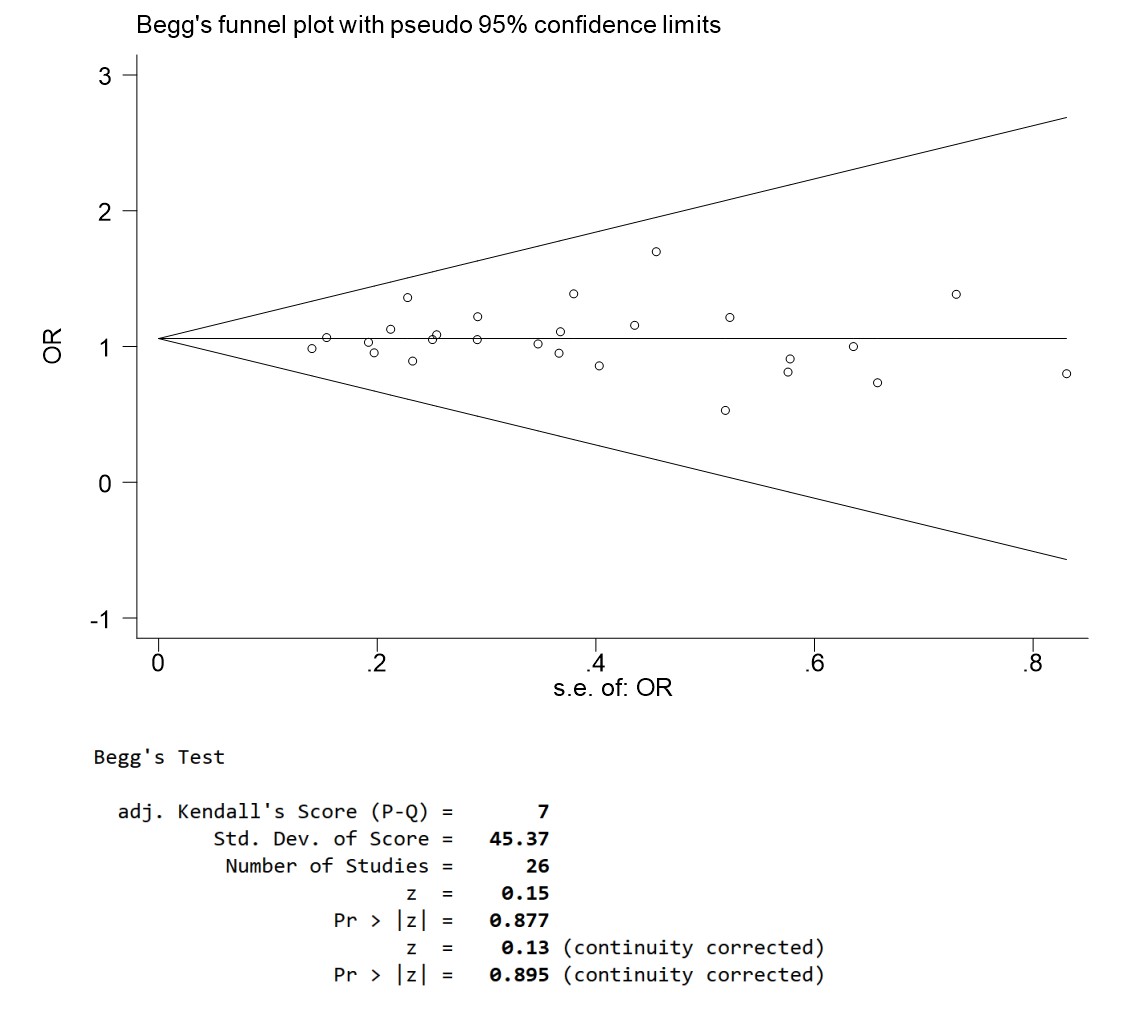


Fig.3.A


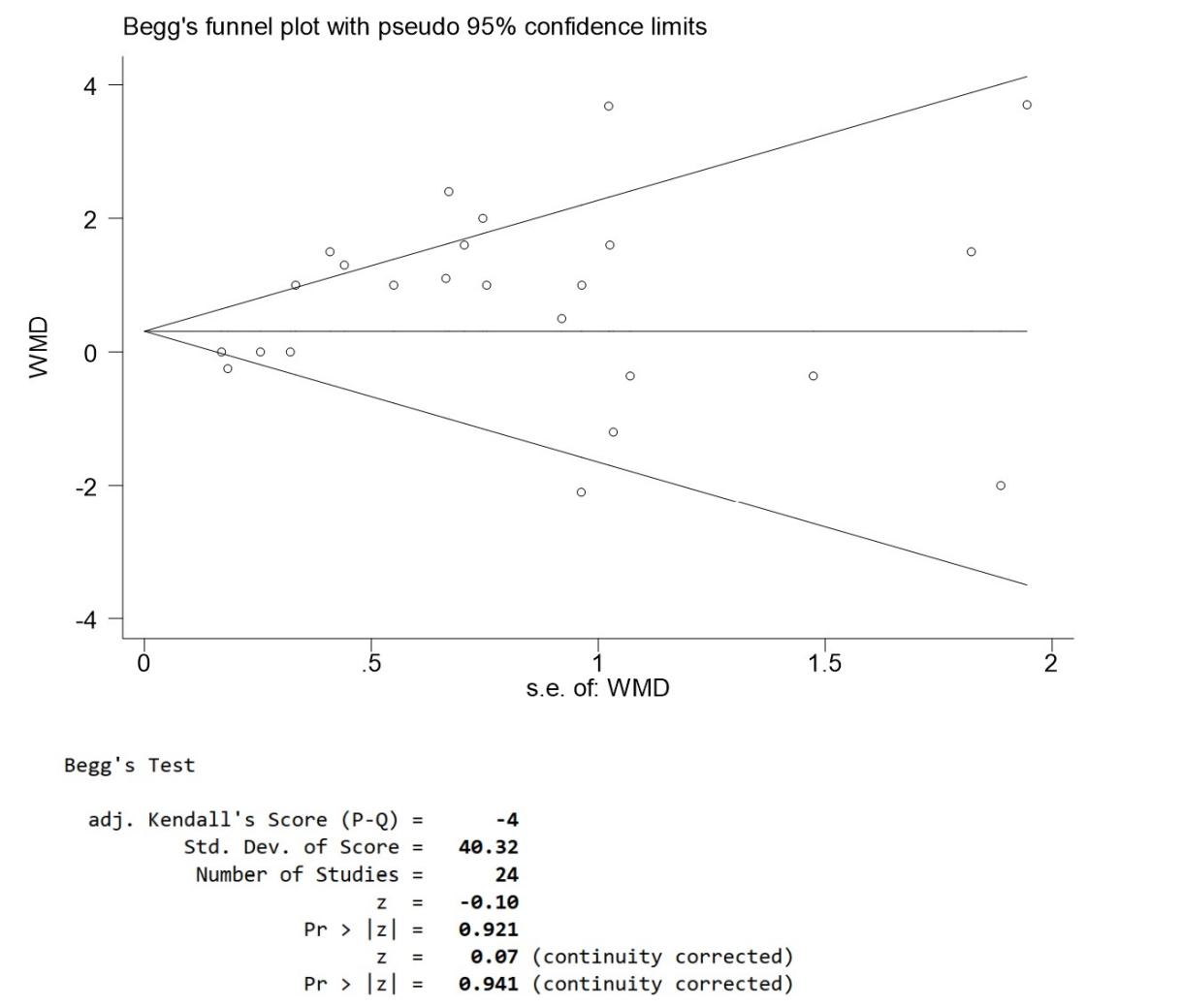


Fig.3.B


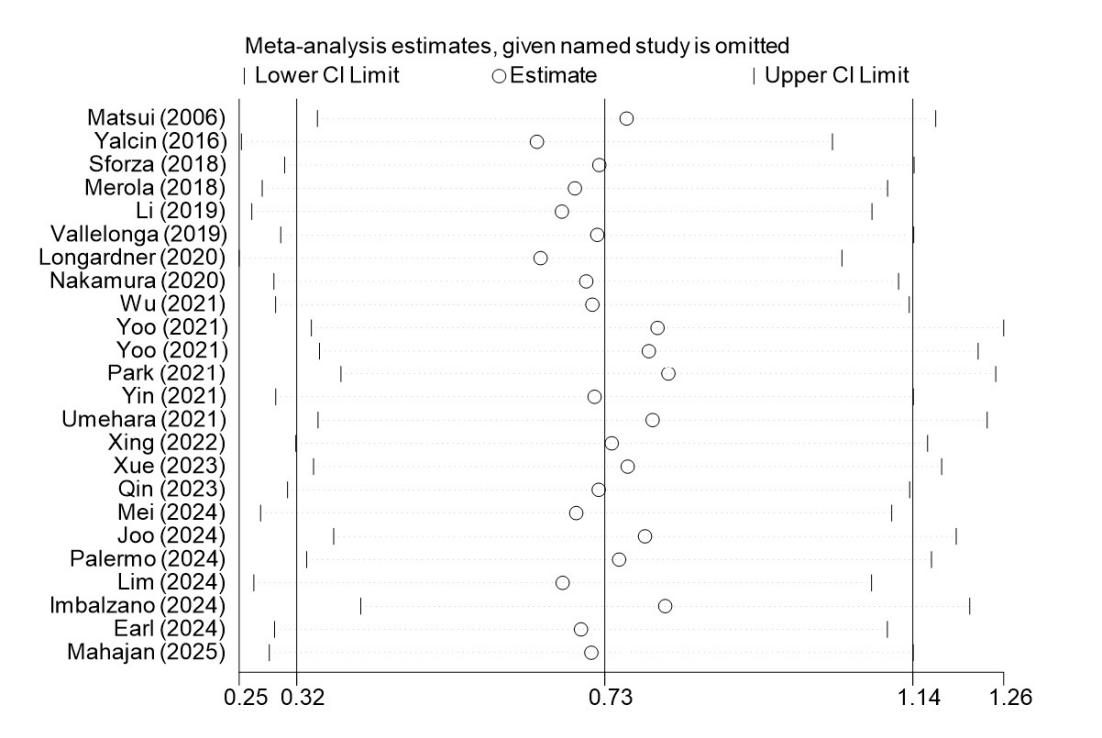


Fig.4.A


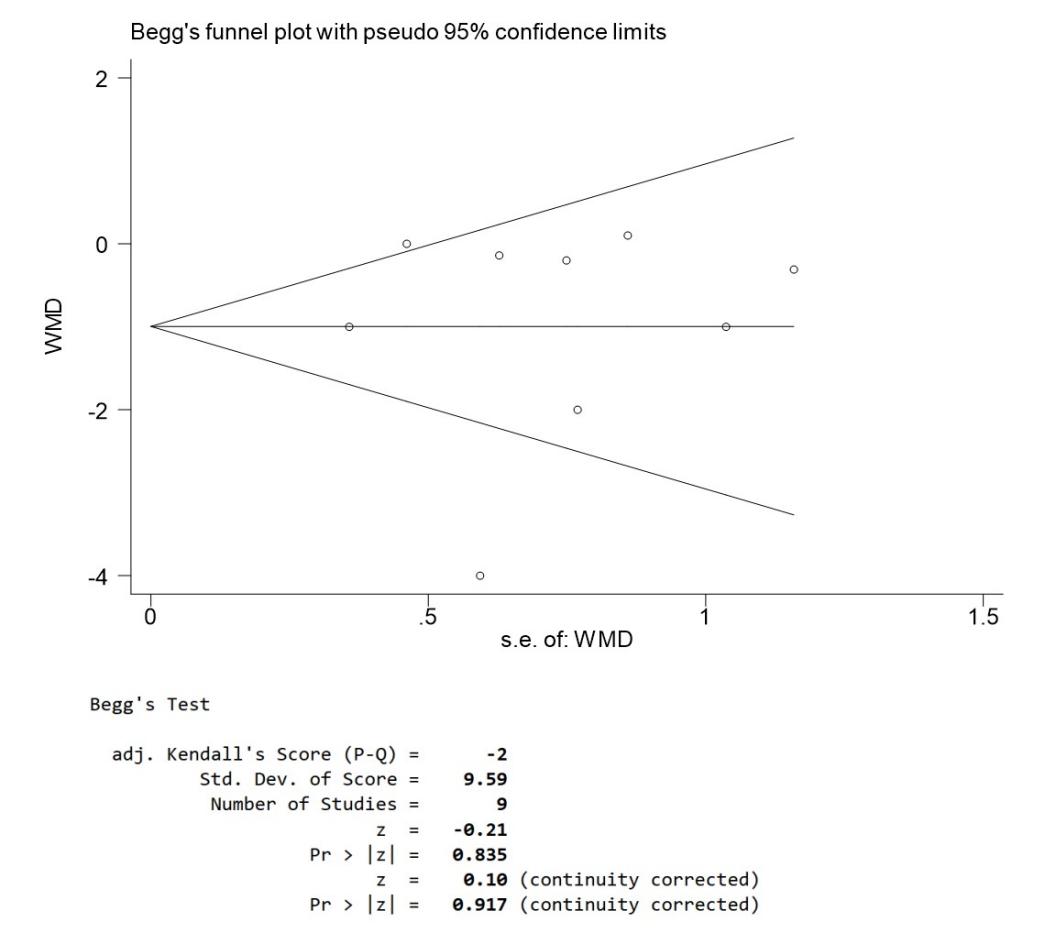


Fig.4.B


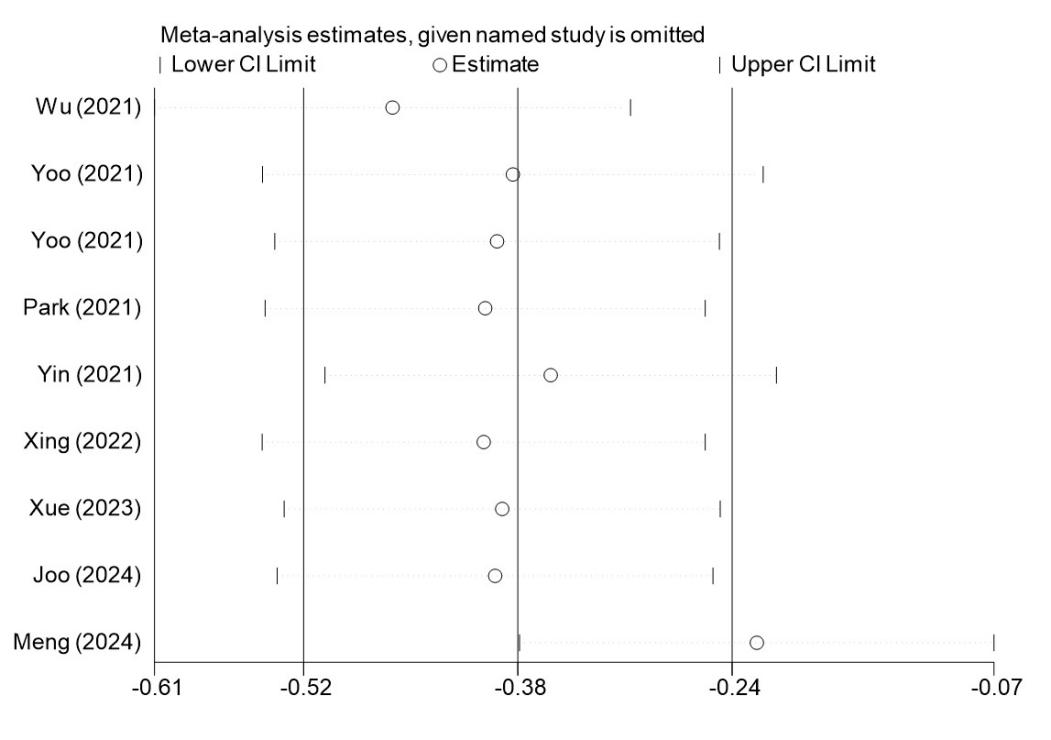


Fig.5.A


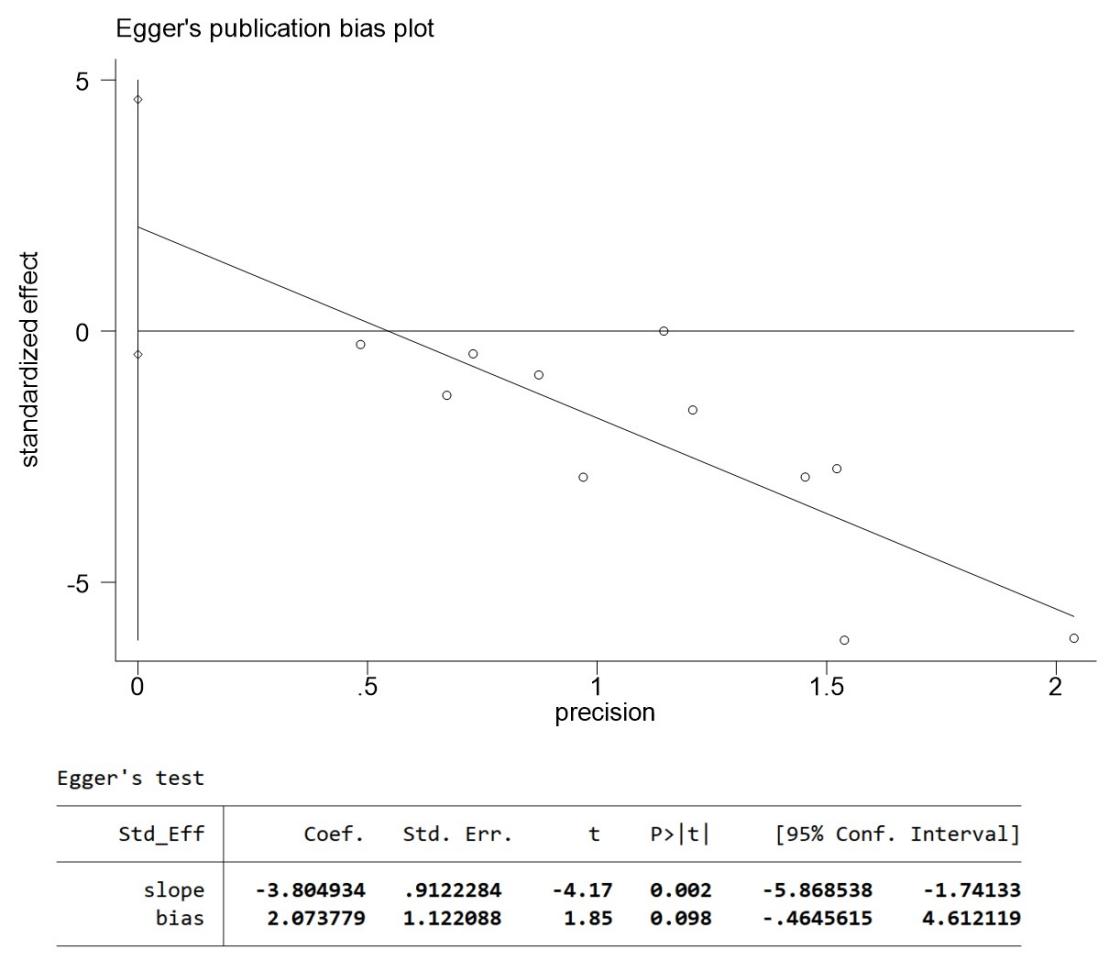


Fig.5.B


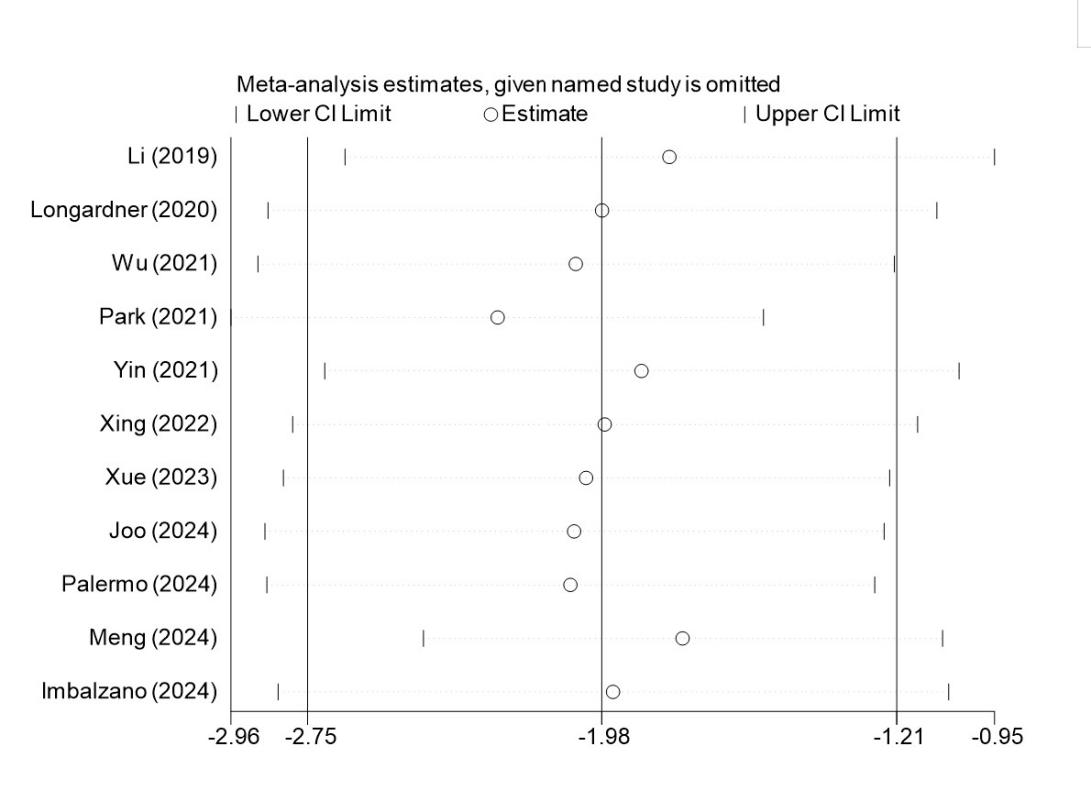


Fig.6.A


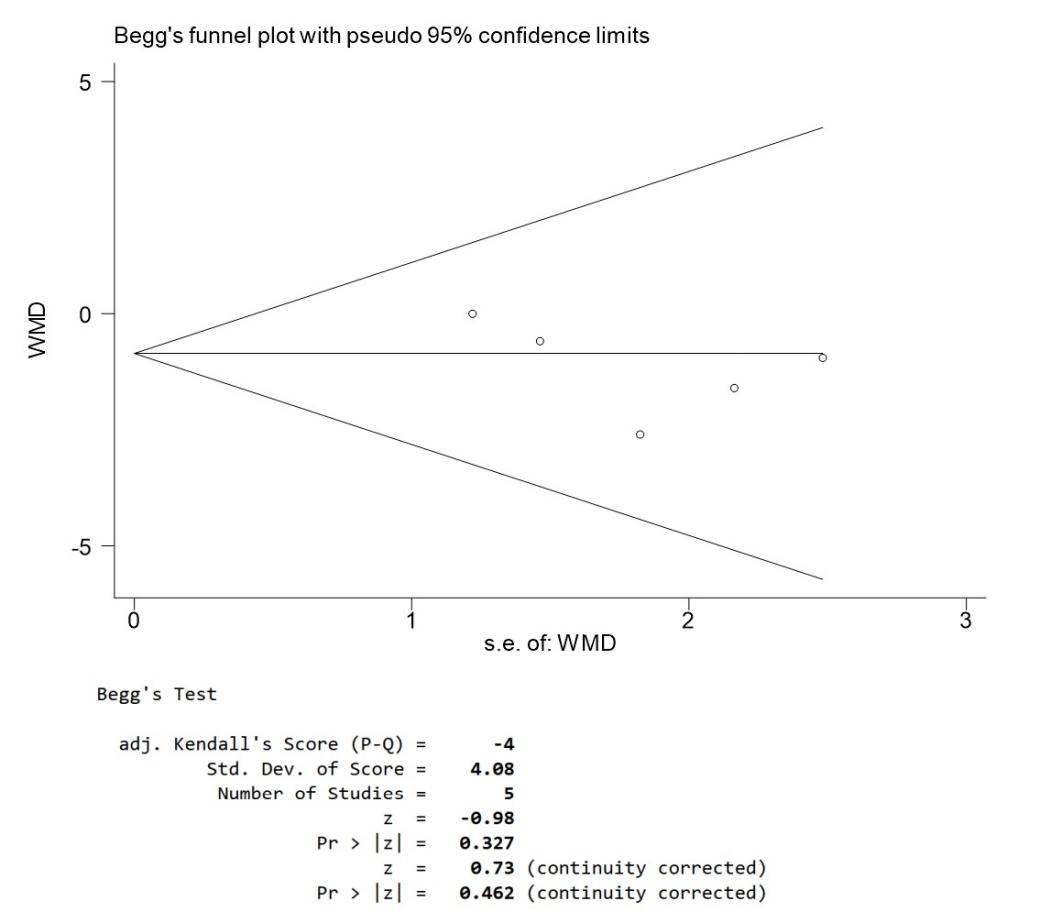


Fig.6.B


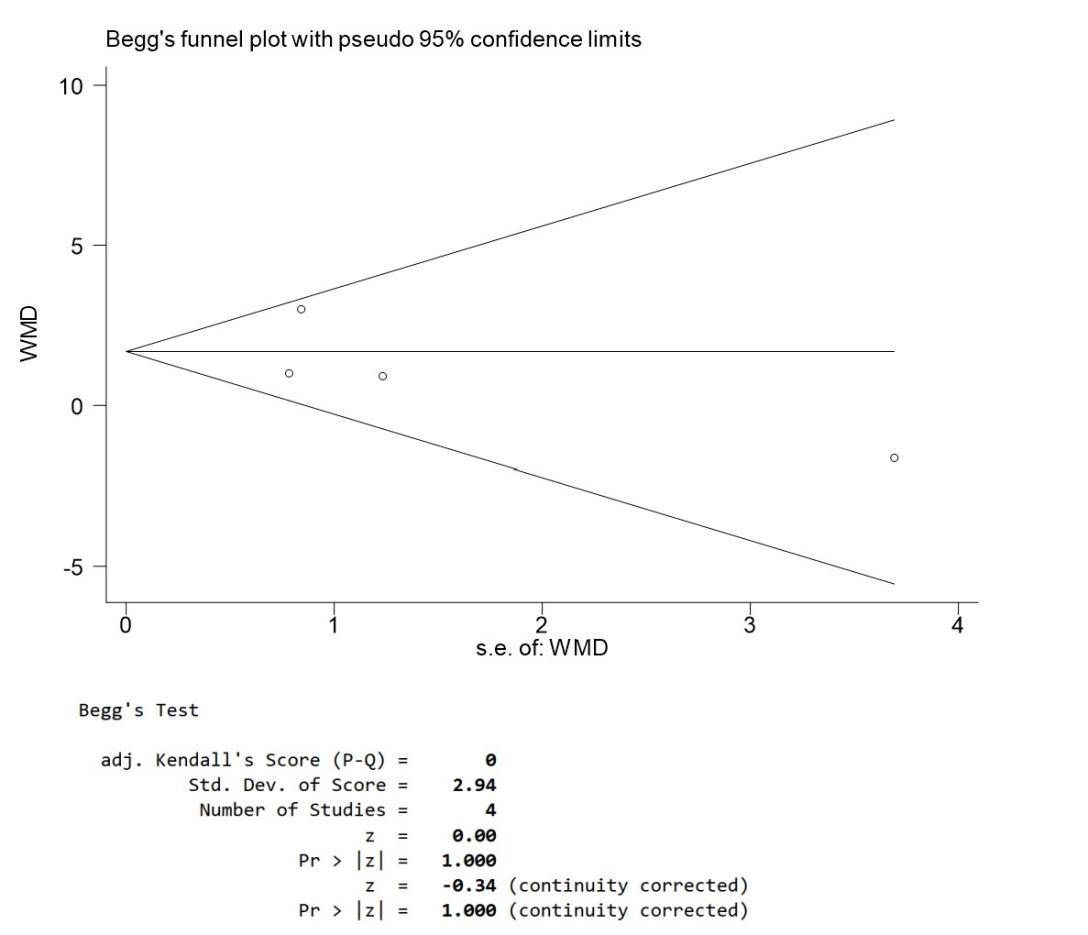


Fig.7.A


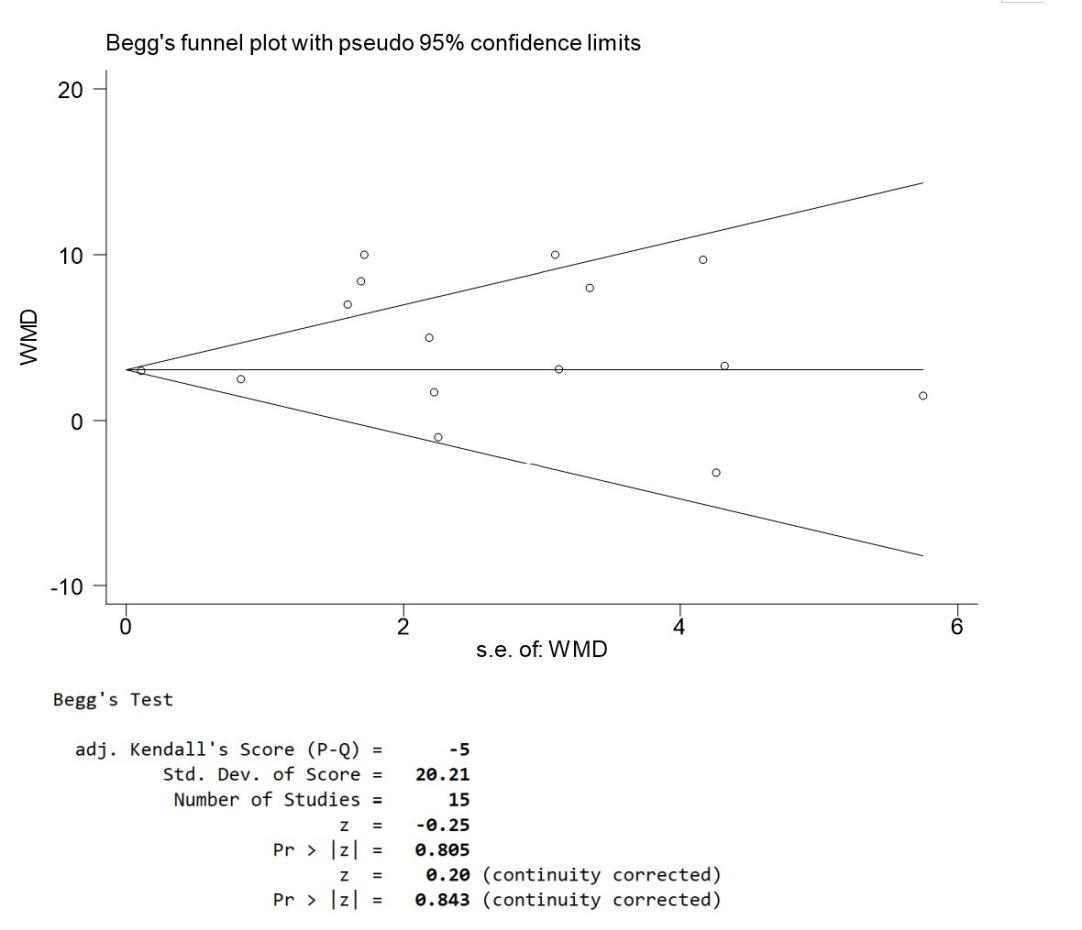


Fig.7.B


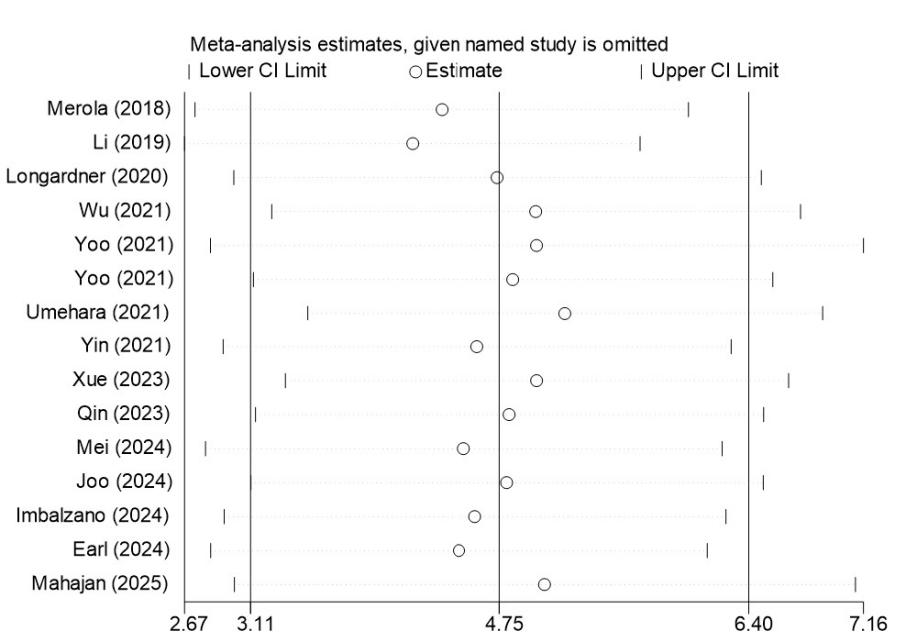

Supplement: Supplementary file 1 [file Data_Sheet_1.docx]
