# Supplementary material for: Orthostatic hypotension in Parkinson’s disease: effects on clinical features and disease severity-a systematic review and meta-analysis
Source: Front Aging Neurosci. 2025 Jul 17;17:1612960. doi: 10.3389/fnagi.2025.1612960 (PMC12310585; doi:10.3389/fnagi.2025.1612960)
Supplement: Supplementary file 2 [file Table_1.docx]

| Searching site | Searching strategy | Number of literature retrieved |
| --- | --- | --- |
| Pubmed | (("Hypotension, Orthostatic"[Mesh]) OR (((Hypotension, Postural[Title/Abstract]) OR (Postural Hypotension[Title/Abstract])) OR (Orthostatic Hypotension[Title/Abstract]))) AND (((((((((((((Idiopathic Parkinson's Disease[Title/Abstract]) OR (Lewy Body Parkinson's Disease[Title/Abstract])) OR (Parkinson Disease[Title/Abstract])) OR (Parkinson's Disease, Idiopathic[Title/Abstract])) OR (Parkinson's Disease, Lewy Body[Title/Abstract])) OR (Paralysis Agitans[Title/Abstract])) OR (Parkinson's Disease[Title/Abstract])) OR (Idiopathic Parkinson Disease[Title/Abstract])) OR (Lewy Body Parkinson Disease[Title/Abstract])) OR (Primary Parkinsonism[Title/Abstract])) OR (Parkinsonism, Primary[Title/Abstract])) OR (Parkinson Disease, Idiopathic[Title/Abstract])) OR ("Parkinson Disease"[Mesh])) | 1275 |
| Embase | ('parkinson disease'/exp OR 'parkinson disease':ab,ti OR 'parkinsons disease':ab,ti OR 'primary parkinsonism':ab,ti) AND ('hypotension, orthostatic':ab,ti OR 'postural hypotension':ab,ti OR 'orthostatic hypotension':ab,ti OR 'hypotension, postural':ab,ti) | 1932 |
| Web of Science | TS=(Idiopathic Parkinson's Disease OR Lewy Body Parkinson's Disease OR Parkinson Disease OR Parkinson's Disease, Idiopathic OR Parkinson's Disease, Lewy Body OR Paralysis Agitans OR Parkinson's Disease OR Idiopathic Parkinson Disease OR Lewy Body Parkinson Disease OR Primary Parkinsonism OR Parkinsonism, Primary OR Parkinson Disease, Idiopathic) AND TS= (orthostatic hypotensionOR hypotension, orthostatic OR postural hypotension OR hypotension, postural) | 443 |
| Cochrane | #1 MeSH descriptor: [Parkinson Disease] explode all trees 6300  #2 ("Parkinson's disease"):ti,ab,kw 13287  #3 (Parkinson Disease):ti,ab,kw 13690  #4 (Primary Parkinsonism):ti,ab,kw 410  #5 #1 OR #2 OR #3 OR #4 13835  #6 (orthostatic hypotension):ti,ab,kw 2147  #7 (postural hypotension):ti,ab,kw 469  #8 (hypotension, orthostatic):ti,ab,kw 2147  #9 (hypotension, postural):ti,ab,kw 469  #10 MeSH descriptor: [Hypotension, Orthostatic] explode all trees 547  #11 #6 OR #7 OR #8 OR #9 OR #10 2376  #12 #5 AND #11 308 | 308 |
